# Dichotomy in redundant enhancers points to presence of initiators of gene regulation

**DOI:** 10.1186/s12864-018-5335-0

**Published:** 2018-12-18

**Authors:** Wei Song, Ivan Ovcharenko

**Affiliations:** Computational Biology Branch, National Center for Biotechnology Information, National Library of Medicine, National Institutes of Health, Bethesda, MD USA

**Keywords:** Redundant enhancers, Gene regulation

## Abstract

**Background:**

The regulatory landscape of a gene locus often consists of several functionally redundant enhancers establishing phenotypic robustness and evolutionary stability of its regulatory program. However, it is unclear what mechanisms are employed by redundant enhancers to cooperatively orchestrate gene expression.

**Results:**

By comparing redundant enhancers to single enhancers (enhancers present in a single copy in a gene locus), we observed that the DNA sequence encryption differs between these two classes of enhancers, suggesting a difference in their regulatory mechanisms. Initiator enhancers, which are a subset of redundant enhancers and show similar sequence encryption to single enhancers, differ from the rest of redundant enhancers in their sequence encryption, evolutionary conservation and proximity to target genes. Genes hosting initiator enhancers in their loci feature elevated levels of expression. Initiator enhancers show a high level of 3D chromatin contacts with both transcription start sites and regular enhancers, suggesting their roles as primary activators and intermediate catalysts of gene expression, through which the regulatory signals of redundant enhancers are propagated to the target genes. In addition, GWAS and eQTLs variants are significantly enriched in initiator enhancers compared to redundant enhancers, suggesting a key functional role these sequences play in gene regulation.

**Conclusions:**

The specific characteristics and widespread abundance of initiator enhancers advocate for a possible universal hierarchical mechanism of tissue-specific gene regulation involving multiple redundant enhancers acting through initiator enhancers.

**Electronic supplementary material:**

The online version of this article (10.1186/s12864-018-5335-0) contains supplementary material, which is available to authorized users.

## Background

Gene regulatory elements such as enhancers establish a spatio-temporal pattern of gene expression in human and other vertebrate genomes. A single vertebrate gene is commonly surrounded by an array of redundant enhancers which often function additively and create a distal, multi-tissue pattern of gene regulation [1]. Multiple redundant enhancers have been identified in the human and mouse genomes and this redundancy acts as not only a regulatory buffer, which prevents deleterious phenotypic effects upon individual enhancer loss, but also as fine-tuning of gene expression [2, 3]. Shadow enhancers, which were originally found in the early Drosophila embryo, are located further away from the target gene and ensure a robust activity matching the primary enhancer [4]. They were reported to be pervasive with one to five copies in more than 60% of examined loci, so that there is no obvious phenotypic changes if one of them is deleted [5]. Large gene loci, which contain multiple non-coding functional elements, such as redundant enhancers, tend to be tissue-specific [6], while housekeeping genes tend to be shorter and experience selective pressure towards compactness [7]. In addition, a recent study also showed that mammalian housekeeping genes, which evolve more slowly than tissue-specific genes [8], also contain fewer enhancers per gene [3]. This variation in locus length may cause bias in functional inference for non-coding elements using gene annotation databases [9]. Although enhancers are frequently located far from their associated genes [10, 11] or sometimes act over an unaffected intermediate gene [12], the proximity between enhancers and transcription start sites (TSSs) of their target genes is critical and reflected in an exponential decay of enhancer-promoter interactions with the increase of the distance [13]. Recent studies of 3D chromatin contact mapping allowed a high resolution profiling of interactions between enhancers and their distantly regulated genes [14, 15], which revealed a hierarchical structure and hub enhancers in a subset of super-enhancers with distinct roles in chromatin organization and gene activation [16].

Tissue-specificity of gene transcription is associated with sequence encryption of enhancers and promoters, as this sequence encryption is reflective of the binding sites of transcription factors (TFs) regulating the target gene and is independent of the distance and orientation between enhancers and genes [17]. Genomic variants in these binding sites might impact and even deactivate enhancer activity in gene regulation [18], which in turn could lead to a disease or disorder [19]. Enhancers that recapitulate tissue-specific gene expression patterns are of continuous interest and various experimental protocols were introduced to predict the activity of tissue-specific enhancers, including chromatin immunoprecipitation sequencing (ChIP-seq) of histone modifications and TFs [1, 20–23]. Using machine learning algorithms such as support vector machines (SVMs) or deep neural networks, one can explore key sequence features and predict enhancers based on the series of consecutive or gapped nucleotides (k-mers) or the TF binding sites (TFBSs) [18, 24–27]. Although the machine learning methods have been used for genome-wide prediction of shadow enhancers [5], they haven’t been used to classify and compare single locus enhancers with redundant enhancers. The loss and gain of single locus enhancers has pronounced effects on the regulatory activity of corresponding genes [28], while the effects of loss of redundant enhancers can be buffered by their duplicates, suggesting that these two enhancer classes might be regulated differently.

We performed a genomic analysis of single and redundant enhancers across nine human tissues and cell lines. We observed that the DNA sequence encryption of single enhancers is distinct from that of redundant enhancers active in the same tissue. This observation allowed us to develop an accurate sequence classifier and identify a set of redundant enhancers, named initiator enhancers, featuring sequence encryption similar to single enhancers. Our results show that single and initiator enhancers are located closer to the nearest TSS and are more evolutionarily conserved than other redundant enhancers. We also demonstrate that initiator enhancers form more chromatin contacts with both nearby TSSs and enhancers, indicating that they may act as primary activators of gene transcription and as intermediate elements establishing regulatory activities between distal enhancers and their target genes. The functional importance of initiator enhancers is further confirmed by overabundance of Genome-wide association study (GWAS) and expression quantitative trait loci (eQTLs) variants within their sequences and an elevated expression level of genes regulated by initiator enhancers.

## Methods

### Definition of single and redundant enhancers

We obtained a set of genome-wide chromatin profiles from the Encyclopedia of DNA Elements (Encode) and Roadmap Epigenomics projects [29, 30], including histone marks, DNase I-hypersensitive sites (DHSs) and TF binding profiles. We selected nine human tissues and cell lines for this analysis, including six (IMR90, GM12878, HMEC, HUVEC, K562 and NHEK; EID: E017, E116, E119, E122, E123 and E127, respectively) with high resolution Hi-C data [15] and three (Brain Hippocampus Middle, Right Ventricle and HepG2; EID: E071, E105 and E118, respectively) that are well-studied [12, 18, 31] (Table 1). We defined tissue-specific active enhancers using the narrow peaks of H3K27ac and H3K4me1 in the corresponding tissue. All consecutive peaks of H3K27ac and H3K4me1 were merged if they overlapped each other. This final merged region was defined as an active enhancer if it contained both H3K27ac and H3K4me1 peaks in the region. Those active enhancers outside promoter regions were selected as candidate enhancers for further categorization. In this study, we defined promoter regions as 1500 base pairs (bps) upstream and 500 bps downstream from a TSS. The TSS and gene locations were retrieved from the UCSC known gene annotation for hg19 [32].

**Table 1:** The performance of classifiers and the fractions of three categories of enhancers for nine tissues used in this study

Encode ID	tissue	auROC	number of enhancers	single (%)	initiator (%)	regular (%)
E017	IMR90 Fetal Lung Fibroblasts	0.90	83,421	1.2	19.4	79.4
E071	Brain Hippocampus Middle	0.91	62,831	2.4	27.4	70.2
E105	Right Ventricle	0.94	83,975	1.5	43.6	54.9
E116	GM12878 Lymphoblastoid	0.84	31,097	4.7	15.1	80.2
E118	HepG2 Hepatocellular Carcinoma	0.8	26,500	5.6	18.6	75.8
E119	HMEC Mammary Epithelial	0.82	42,674	3.3	17.0	79.7
E122	HUVEC Umbilical Vein Endothelial	0.80	35,073	4.3	21.8	73.8
E123	K562 Leukaemia	0.86	27,565	5.3	20.4	74.4
E127	NHEK-Epidermal Keratinocyte	0.83	43,701	3.0	16.8	80.2

Each gene locus was defined as a region that extends from the current gene to the nearest gene in both directions along the genome, which results in a pair of neighboring gene loci overlapping each other. A candidate enhancer was denoted as a single enhancer when it was 1) a single intronic enhancer associated with the host gene or 2) a single intergenic enhancer for both flanking genes. If there were multiple enhancers located in a gene locus, all of them were categorized as redundant enhancers. Finally, an enhancer was defined as a 400 bps long DNA segment, represented by an extension of 200 bps in both directions along the genome from the central position of the candidate enhancer. For all tissue-specific active enhancers, only those containing less than 30% repetitive sequences were retained in our study to ensure a reliable sequence-based analysis.

### Training of classifier and predicting initiator enhancers

We first characterized enhancer DNA sequences by their density of all 6-mers. Given a DNA sequence, the density of a 6-mer was calculated as the occurrence of the 6-mer divided by the length of the non-repetitive part of that sequence. Based on these sequence features, we built support vector machine (SVM) models to identify single enhancers from the genomic background, and later to separate initiator enhancers from regular enhancers. Our SVM models used LIBSVM [33] with a Gaussian kernel (svm-train -t 2 -b 1 -w1 5 -w-1 1). The single enhancers with the top 25% strongest signal (averaging the total strength of overlapped peaks in that enhancer region) were selected as positive training samples. We generated five control sequences by randomly sampling the human genome sequences and matching the length and repeat-content to each enhancer sequence from the positive set. We also excluded all candidate enhancer regions in the corresponding tissue, transcribed enhancers reported in CAGE [34] and VISTA enhancers [35] from our control sequence generation. We used a five-fold cross validation to evaluate the performance of our classifiers. We applied the classifier to redundant enhancers to predict those initiator enhancers which feature the same sequence encryption as single enhancers, with a False Positive Rate (FPR) of 5%.

### Proximity to TSSs and evolutionary conservation

The central point of an enhancer was used to represent the position of this enhancer for calculating distances and Hi-C contacts. The TSS for an intronic enhancer was the TSS of the host gene, while the nearest TSS of an intergenic enhancer was defined as the closest TSS of its two neighboring genes. We evaluated the phastCons alignment score [36] at the nucleotide level and the average score for each enhancer sequence was calculated. The phastCons 46way placental wig files were downloaded from the UCSC genome browser [32] and only non-repetitive regions of the enhancers were evaluated. The background conservation data are based on 10x random genomic regions located at the same distance to randomly selected genes as the corresponding enhancers in each class to their nearest genes to control for distance to the TSS.

### Chromatin contacts

Hi-C data from six human cell lines with a 5 kilobases (kb) resolution (IMR90, GM12878, HMEC, NHEK, K562 and HUVEC) were retrieved from Rao’s work (GSE6352) [15]. Knight-Ruiz Matrix Balancing (KR) [37] and Benjamini-Hochberg FDR controls [38] were used to correct for the multiple testing hypotheses (FDR rate = 0.1), as suggested in Rao’s work. Chromatin contacts longer than 1 megabase (Mb) were not considered. The background count of chromatin contacts is based on 10x randomly selected pairs of genomic regions located at the same distance as the distance from the corresponding enhancer to its target (to control for distance effects).

### TFBSs enrichment and histone mark signal intensities in different classes of enhancers

We took advantage of the available ChIP-seq TFBS data to calculate the TFBS enrichment of enhancers in HepG2, GM12878 and K562 cell lines [29, 32]. In the corresponding cell line, we compared the TFBSs enrichment between single (positive set) and redundant (control set) enhancers, and between initiator (positive set) and regular (control set) enhancers, respectively. The number of overlapping regions between positive or control sequences and ChIP-seq peaks of a particular TFBS was added and averaged by the total length of either positive or control sequences, respectively, to compute the frequency of TFBSs. Fold-enrichment of a TFBS was then computed as a ratio of its frequency in the positive set to that in the control set. A *p*-value was calculated using the Fisher’s exact test and only TFBSs with the *p*-value < 0.05 and fold enrichment > 1.5 were included into the analysis. Similarly, for the analysis of a particular histone mark, the signal intensities of overlapping ChIP-seq peaks were averaged by the number of enhancers in both positive and control sets, followed by a fold-enrichment and *p*-value calculation.

### Density of GWAS and eQTLs variants

The GWAS Catalog data were downloaded from NHGRI-EBI [39] and GTEx eQTLs v7 data were obtained from the GTEx Portal (www.gtexportal.org) for the variant density analysis. The density of variants was calculated as the number of variants falling into genomic regions occupied by enhancers from a particular class over the total number of enhancers in that class.

## Results

### Sequence classification of single, initiator and regular enhancers

Although widespread redundant enhancers have been previously reported in many comprehensive studies and linked to phenotypic robustness [2–5], the mechanisms and evolutionary stability of the single enhancer regulatory programs remain to be studied in detail [28]. In this study, we focused on comparing and contrasting single and redundant enhancers, and the regulatory mechanisms employed by them. We selected nine human tissues and cell lines for this analysis and refer to these tissues and cell lines as tissues for simplicity (See Methods). Among all these tissues, IMR90 and the right ventricle have the largest number of enhancers (over 83,000), while HepG2 contains the smallest number of enhancers (about 26,000). The percentage of single enhancers among all enhancers in a particular tissue ranges from 1.2% in IMR90 to 5.6% in HepG2, with an average of 3.5% (Table 1). On average, we observed that 38% of gene loci contain two or more enhancers, 15% of loci contain a single enhancer and the remaining 47% of loci have no enhancers and these percentages vary across different tissues. In addition, 7% of gene loci have more than 10 enhancers in the same locus, with the maximum of 14% for IMR90 and the minimum of 3% for HepG2 and K562, respectively, suggesting a non-negligible amount of gene loci packed densely with enhancers (Additional file 1: Figure S1).

**Fig. 1:**
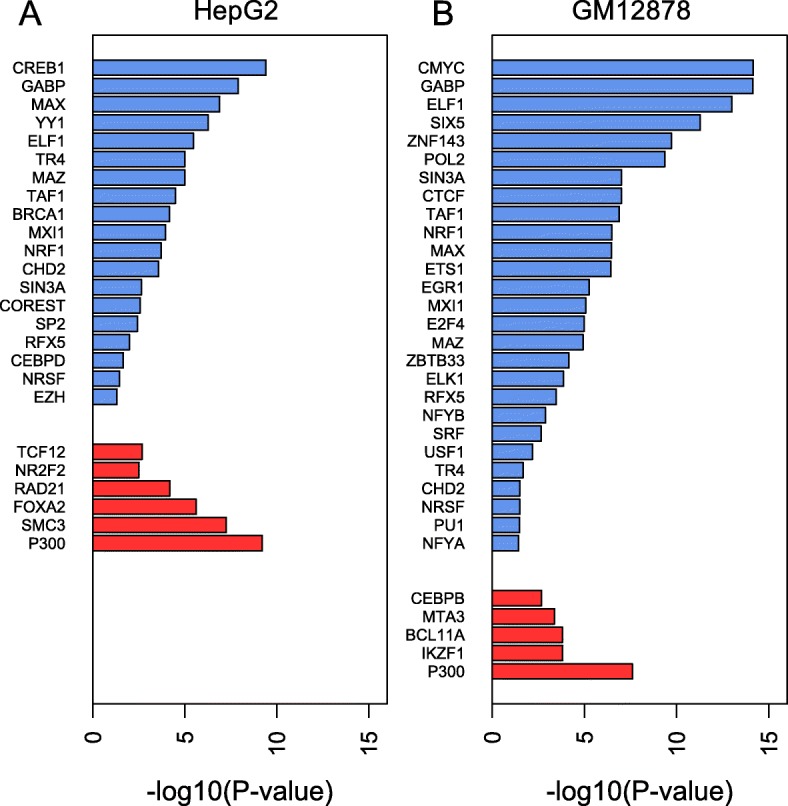
Enrichment of TFBSs in single enhancers compared to redundant enhancers, in (**a**) HepG2 and (**b**) GM12878 cell lines. Blue color shows TFBSs enriched in single enhancers, while red color shows TFBSs enriched in redundant enhancers. *P*-value was calculated using the Fisher’s exact test. Only TFBSs with the *p*-value < 0.05 and enrichment fold > 1.5 are shown

We focused on the difference in genomic encryption between single and redundant enhancers, in terms of their composition of TFBSs, to study if these two classes of enhancers are associated with different gene regulatory mechanisms. In the cases of HepG2, GM12878 and K562 cell lines with ChIP-seq TFBS data, we compared the enriched TFBSs between these two classes of enhancers (Fig. 1**,** Additional file 1: Figure S2A). In redundant HepG2 enhancers, the binding sites of TR4, GABP, SP2 and NRF1 are depleted and the binding sites of FOXA2, P300 and NR2F2 are enriched as compared to single enhancers. In redundant GM12878 enhancers, the binding sites of GABP, ZBTB33, ETS1, E2F4 and SIX5 are depleted and the binding sites of C/EBPβ and MTA3 are enriched as compared to single enhancers. FOXA2 and NR2F2 are known to play critical roles in liver development and biological functions [40, 41] and C/EBPβ and MTA3 are involved in the development or differentiation in B cell Lymphocytes [42–44]. In contrast, transcription factors ZBTB33, ETS1, E2F4, SIX5 in GM12878 and TR4, SP2 and NRF1 in HepG2, which are enriched in single but depleted in redundant enhancers, are associated with repressor functions in gene expression [45–51]. In K562, the transcription factor E2F6, which might function as a repressor, is enriched in K562 single enhancers, while transcription factors STAT5, TAL1 and GATA2, which are all associated with growing culture and differentiation of leukemia cell, are significantly enriched in redundant enhancers [52–55] (Additional file 1: Figure S2A). These results indicate that single enhancers are enriched for a particular set of TFBSs, which are different from those in redundant enhancers and may not necessarily function as key factors of tissue-specificity, while redundant enhancers are specifically associated with tissue-specific biological processes. This difference between single and redundant enhancers reveals that distinct functional mechanisms are associated with these two classes of enhancers, suggesting that enhancer duplicity is not simply a source for robustness of transcription but also a reflection of different regulatory rules.

**Fig. 2:**
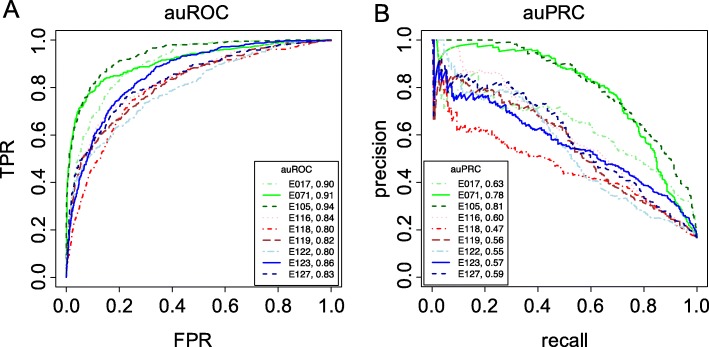
Classification accuracy for single enhancers vs randomly sampled human genomic sequences. **a** The receiver operating characteristic (ROC) and (**b**) the precision recall (PRC) curves for nine tissues

This enrichment of particular TFBSs in single enhancers indicates a distinct biological mechanism of self-sustained gene regulation and suggests a possibility that some loci of redundant enhancers might operate in a hybrid mode, which employs self-sustaining enhancers similar to single enhancers (which we named initiator enhancers) that are surrounded by other redundant enhancers. This hybrid mode of regulation would benefit from reliable transcriptional activation by an initiator enhancer and evolutionary stability introduced by redundant enhancers. To identify such cases of hybrid regulation, we used a classifier based on the Support Vector Machine (SVM) and 6-mer DNA sequence representation to define the DNA sequence encryption of single enhancers, which was subsequently used to detect initiator enhancers within redundant enhancers (See Methods). Our SVM classifier showed a consistently high accuracy in differentiating single enhancers from random genomic sequences across nine tissues. The overall averaged area under the receiver operating characteristic curve (auROC) using a five-fold cross validation was 0.85 (Fig. 2), with right ventricle displaying the highest accuracy with the auROC of 0.94, and HepG2 having the lowest auROC of 0.80. A similar trend was observed for the area under the precision-recall curve (auPRC), with the maximum value of 0.81 for right ventricle and the minimum value of 0.47 for HepG2. Our results show that a small fraction of redundant enhancers (initiator enhancers) shares the genomic encryption with single enhancers and is different from regular enhancers. On average, 22% of total enhancers are classified as initiator enhancers, with the maximum fraction of 44% in right ventricle and the minimum fraction of 15% in GM12878 (Table 1). Although single enhancers only represent a small part of the total enhancers, they self-sustain the gene regulatory program in a locus with a particular set of TFBSs. Initiator enhancers which feature a genomic encryption different from the remaining redundant enhancers (referred to as regular enhancers) may introduce self-sustainability of transcriptional regulation into the loci of redundant enhancers. Enrichment analysis indicates that the set of enriched TFBSs in initiator enhancers is not only similar but also larger than the set of single enhancer TFBSs when both of them are compared to redundant enhancer TFBSs. The majority of TFBSs feature a greater enrichment and significance in initiator enhancers than in regular enhancers, indicating a homogeneous sequence composition of initiator enhancers (Additional file 1: Figure S2B).

### Single and initiator enhancers are closer to genes and more evolutionarily conserved than regular enhancers

To explore functional characteristics of the three classes of enhancers, we first compared their gene ontology (GO) enrichment as quantified using the tool named GREAT [56], for the right ventricle and HepG2—two tissues involved in distinct biological pathways. Our results show that in both tissues single enhancers are mainly involved in metabolic, biosynthetic and catabolic functions, which are associated with housekeeping genes. Redundant enhancers, however, are more tissue-specific and are associated with multiple cell development and differentiation processes (Additional file 1: Figure S3). For example, in the right ventricle, the genes proximal to redundant enhancers are related to the mechanistic and response functions of the heart, such as regulation of heart contraction, response to oxygen levels, response to hypoxia, regulation of cardiac muscle contraction and striated muscle cell development. About 15 processes are directly related to cardiac functions, while the rest are related to energy, kinase activity, signaling pathway, carbohydrate and glucose metabolic processes (Additional file 1: Fig. S3A). In HepG2, the functions of genes associated with redundant enhancers include liver functions, such as liver development, hepaticobiliary system development, and metabolic processes of alcohol, phospholipid, lipid, steroid, glycerophospholipid, cholesterol, glucose (Additional file 1: Fig. S3B). We didn’t observe a noticeable difference between initiator and regular enhancers in their associated biological processes as this GO analysis is based on flanking genes, while initiator and redundant enhancers are flanking the same genes by definition (with the exception of some loci containing only redundant enhancers that miss initiator enhancers). The fact that single enhancers are highly associated with housekeeping genes and involved in similar fundamental biological processes across different tissues suggests their indispensable roles in regulatory activities.

**Fig. 3:**
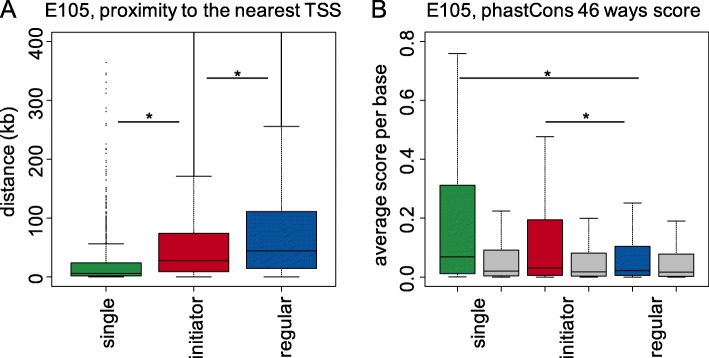
**a** Distribution of distances between enhancers and their nearest TSSs. **b** Distribution of phastCons conservation scores for different classes of enhancers. Single and initiator enhancers are more evolutionarily conserved than regular enhancers. The plot shows the analysis performed for the right ventricle (E105) tissue. Grey color means background value. (* - *p*-value < 0.01). *P*-value was calculated using the Wilcoxon rank-sum test

Single enhancers are located in more compact gene loci as compared to redundant enhancers. The average length of a single enhancer locus is 160 kb, while the average length of a locus populated by redundant enhancers is 254 kb. Although we are not rejecting the hypothesis that compactness of a locus might be the selective evolutionary force limiting the number of enhancers in a locus, we note that 95.4% of single enhancer loci are longer than 10 kb and thus provide sufficient genomic space for multiple additional enhancers. We propose that there is a selective pressure for expansion of the loci with redundant enhancers, as the genomes of higher vertebrates are constantly expanding and the appearance of either a new gene or a chromosomal break separating some of the redundant enhancers from their target genes might have detrimental effects on the fitness of the species, thus the loci of redundant enhancers might expand faster than the genome. In concordance with this hypothesis, we calculated, across the nine tissues, the average distance from single, initiator and regular enhancers to their nearest TSS, which is 28 kb, 68 kb and 103 kb (Fig. 3a, Additional file 1: Figure S4) and the average size of the loci containing these classes of enhancers is 160 kb, 254 kb and 267 kb (Additional file 1: Figure S5), respectively. The difference between loci with initiator and regular enhancers is due to the presence of loci that contain regular enhancers exclusively and the average length of such loci is 303 kb. In addition, 70% of all enhancers in our study are intronic and 30% are intergenic. For single enhancers the fraction of intronic enhancers is 82%. Moreover, 41% of single enhancers and 34% of both initiator and regular enhancers are within the first intron of a gene—a region known to harbor key regulatory elements [57–60]. Single and initiator enhancers are much closer to the nearest TSS than regular enhancers, suggesting their potential functions as primary enhancers in transcriptional regulation compared to the distant regular enhancers as secondary (or shadow) enhancers [4].

In general, regulatory elements involved in similar biological functions and pathways tend to experience a similar selective pressure [61]. As single enhancers are associated with similar biological processes across different tissues, populate compact gene loci and establish transcriptional regulation of a target gene lacking a functional backup due to the absence of redundant enhancers, we speculated that they are evolving under a stronger evolutionary constraint. To assess selective constraints acting on the three classes of enhancers, we used the phastCons evolutionary conservation scores derived from 46 placental mammal sequence alignments [36]. For 8/9 tissues, single and initiator enhancers are significantly more conserved than regular enhancers (Fig. 3b**,** Additional file 1: Figure S6). In the case of HepG2, the difference of conservation levels between initiator and regular enhancers are small and not that significant (*p*-value = 0.37), which might be caused by its low performance classifier noted previously. Across all tissues, single enhancers have the highest average conservation score, followed by initiator and regular enhancers. The strongest sequence constraint on single enhancers suggests their indispensability in gene regulation and is consistent with the stronger evolutionary constraint of their potential target genes, the housekeeping genes, which evolve slower than tissue-specific genes [8]. Initiator enhancers, which demonstrate a significantly higher level of sequence conservation than regular enhancers (*p*-value < 2.2 × 10^− 16^, Wilcoxon rank sum test), are likely to play an important role in regulation of tissue-specific genes and to be supported by secondary (regular) enhancers that results in the establishment of a complex regulatory profile of gene expression.

### Initiator enhancers feature chromatin contacts with both promoters and regular enhancers

**Fig. 4:**
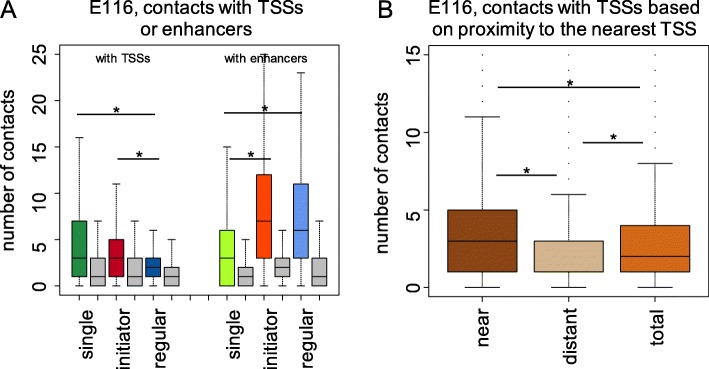
**a** Distribution of number of contacts between three classes of enhancers and TSSs/other enhancers with 1 Mb cutoff distance for interactions. **b** Distribution of number of contacts between enhancers and TSSs according to their distances to the nearest TSS (near half, distant half and total enhancers) with 1 Mb cutoff distance for interactions. The plot shows the analysis performed for the GM12878 (E116) cell line. (* - *p*-value < 1 × 10^− 7^). *P*-value was calculated using the Wilcoxon rank-sum test

Initiator enhancers are redundant enhancers but distinguishable from the regular enhancers by their distinct evolutionary and genomic properties. They are in a closer proximity to the genes and more evolutionarily conserved, which makes them critical blocks for regulatory activity in a locus containing multiple enhancers. We took advantage of the available Hi-C data with high resolution (5 kb) available for six tissues [15] and focused on the number of contacts formed by each enhancer (Fig. 4a**,** Additional file 1: Figure S7). Although the average number of contacts is different among these tissues, they all feature a similar trend that single and initiator enhancers have approximately twice as many contacts with nearby genes as regular enhancers, suggesting their important functions of direct regulation of genes. Meanwhile, initiator and regular enhancers feature almost twice as many interactions with nearby enhancers as single enhancers, suggesting a highly connected enhancer network formed by redundant enhancers.

The difference in average contact numbers among the three classes of enhancers suggests different gene regulatory modes for each class: 1) single enhancers have a high level of direct interactions with nearby genes but fewer interactions with other enhancers, reflecting their self-sustainable gene regulatory activity; 2) initiator enhancers maintain a high level of contacts with both nearby genes and other enhancers, indicating their central position in enhancer networks and a critical role of acting directly on their target genes and propagating regulatory signals of regular enhancers; 3) regular enhancers, which represent the majority of all enhancers, form a high level of enhancer-enhancer interactions but a relatively low level of direct enhancer-TSS interactions. We also observed that initiator enhancers maintain a significantly larger number of both enhancer-promoter and enhancer-enhancer contacts across different topologically associating domain (TAD) regions than regular enhancers (Additional file 1: Figure S8), revealing an ability of initiator enhancers to partake in distal gene regulation and to connect regular enhancers to their distal target genes.

Our analysis shows that enhancer clusters formed by regular enhancers are strongly dependent on the presence of intermediate initiator enhancers connecting them and their target genes. In support of this hypothesis of a general regulatory signal propagation through initiator enhancers, we observed a 2.0-fold enrichment of enhancer-TSS contacts for the half of redundant enhancers closest to the nearest TSS within 1 Mb distance cutoff versus the more distant half (*p*-value < 2.2 × 10^− 16^, Wilcoxon rank sum test) (Fig. 4b**,** Additional file 1: Figure S9). This is consistent with the previous analysis of proximity showing that initiator enhancers are located much closer to the nearest TSS than redundant enhancers. Although this hierarchical structure of enhancer collaboration has already been observed in super-enhancers [16, 62], according to our results, this mechanism of signal propagation from distant regular enhancers to the target genes through the intermediate initiator enhancers might be a common rule for gene regulation rather than being limited to super-enhancers. Additionally, among all the enhancers from each class that maintain chromatin contacts, on average 85% of single enhancers form interactions with nearby TSSs while this fraction decreases to 64% for initiator and 55% for regular enhancers, suggesting that a major role of single enhancers is in activating gene regulation directly. Meanwhile, a much larger fraction of initiator and regular enhancers than single enhancers maintains interactions with nearby enhancers (Additional file 1: Figure S10A, B). In concordance with these observations, the fraction of initiator enhancers interacting with both TSSs and other enhancers is the highest among all three classes. For the CTCF and cohesin factors RAD21 and SMC3, which are important for forming 3D genomic structures, their relative enrichment is much higher in initiator than regular enhancers (Additional file 1: Figures S2B and S10C). In addition to the higher level of enrichment of looping factors, the overall higher enrichment of TFBSs in initiator enhancers than single and regular enhancers may also indicate their role in contacting both promoter and regular enhancers through involved TFs. However, single and regular enhancers also show complementary ability to interact with both target genes and nearby enhancers, although at a reduced rate, which implies a complexity of the human gene regulation landscape.

### Initiator enhancers are strongly associated with gene expression changes and human disease variants

After showing that initiator enhancers feature unique genomic characteristics distinguishing them from regular enhancers, we focused on their functional importance in transcriptional events. Since the epigenetic marks, including histone modifications and DNA methylation, are reflective of fundamental regulatory events [63–67], we quantified the enrichment of available ChIP-seq histone marks for the three classes of enhancers: contrasting single and regular enhancers and contrasting initiator and regular enhancers across different tissues, respectively (Additional file 1: Figure S11). Single enhancers demonstrate an enrichment in TSS-proximal histone marks (H3K4me2 and H3K4me3), which reflects their proximity to their target genes. Initiator enhancers, on the other hand, display an additional strong enrichment in the marks specific to active enhancers—H3K27ac and H3K4me1—when compared to regular enhancers. This further supports our finding that initiator enhancers represent the key and most active subclass of enhancers. To verify that the initiator enhancers are crucial for gene regulation and to study how their activity affects gene expression, we used RNA-seq expression data for four categories of genes neighboring different classes of enhancers: 1) connected with single but not with initiator enhancers, 2) connected with initiator but not with single enhancers, 3) connected with regular only but not with single or initiator enhancers, 4) no connections with enhancers (control set). Our results show that genes that feature Hi-C interactions with initiator enhancers have a significantly higher expression level than those connected only to regular or single enhancers, suggesting a functional importance of initiator enhancers in recruiting regular enhancers and elevating the expression level of target genes (Additional file 1: Figure S12).

**Fig. 5:**
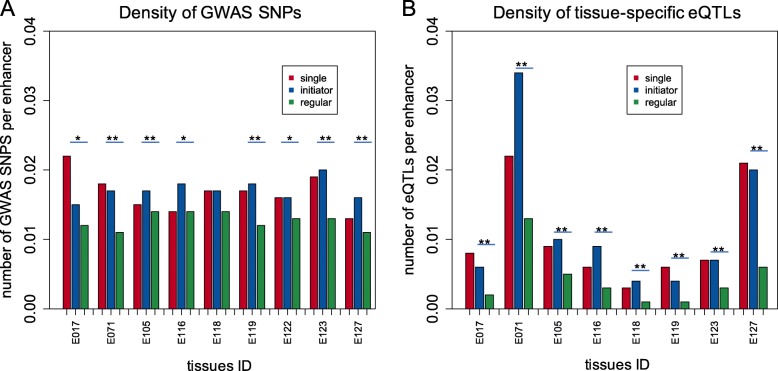
Density of (**a**) GWAS and (**b**) eQTLs variants in three classes of enhancers for different tissues. (* - *p*-value < 0.05,** - *p*-value < 1 × 10^− 4^). *P*-value was calculated using the Binomial test

To further address the essential role of initiator enhancers in transcriptional events and address the phenotypic consequences of disrupting their stability, we examined the overlap between these three classes of enhancers and the human disease and eQTLs variants. Our result shows that the average density of both GWAS and eQTLs variants is significantly higher in initiator enhancers than that in regular enhancers (Fig. 5). This validates and strengthens our previous analysis that initiator enhancers maintain a crucial function in gene regulation and mutations of their sequence may lead to a change in target gene expression and subsequently lead to a disease phenotype.

## Discussion

Human genes usually employ multiple enhancers in their loci to establish transcription robustness and evolutionary stability. In this work, we separated tissue-specific enhancers into three classes according to the number of enhancers in the corresponding gene locus and their genomic sequence encryption. We demonstrated that each class of enhancers shows specific characteristics that are associated with their distinct roles in transcription and different gene regulatory mechanisms. Single enhancers, which represent the only enhancer existing in a gene locus, are different from redundant enhancers not only because of their lack of backup enhancers, but also because of their proximity to nearby genes and evolutionary conservation greater than in redundant enhancers, as well as GO enrichment showing their strong association with housekeeping genes. A subset of the top TFBSs enriched in single but depleted in redundant enhancers is associated with repressors. All these results suggested that single enhancers perform multiple types of regulatory activity, while in the loci of redundant enhancers these functions of enhancing and repressing of transcription are distributed between multiple enhancers and silencers. An elevated level of chromatin contacts between a single enhancer and its target TSS suggests a direct regulation of target genes by single enhancers, while a low level of contacts between them and other enhancers indicates their ability to fulfil biological functions in an independent manner.

There is a specific subclass of redundant enhancers called initiator enhancers that are different from regular enhancers based on their DNA sequence similarity to single enhancers. Initiator enhancers are located closer to the nearest genes and are more evolutionarily conserved than regular enhancers. Although the two classes of enhancers are involved in similar tissue-specific biological processes (as their loci largely overlap), they have notable differences in forming chromatin contacts with nearby genes. Initiator enhancers feature twice as many contacts with TSSs of nearby genes as regular enhancers, suggesting their role as activators of gene regulation. The fact that initiator enhancers form a large number of contacts with both genes and other enhancers makes them potential intermediate catalysts responsible for collecting transcriptional signals from a cluster of regular enhancers and transmitting these signals to target genes. Strong enrichment of GWAS and eQTL variants and an elevated level of gene expression associated with initiator enhancers also suggest their key role in gene regulation compared to regular enhancers. Although this hierarchical structure of multiple enhancers has also been observed in super-enhancers, a large fraction of the redundant enhancers in our study are not super-enhancers. For example, in K562, about 4.1% of single, 13.0% of initiator and 11.7% of regular enhancers overlap with identified super-enhancers [68]. However, in HepG2, these fractions drop to 1.3%, 4.1% and 4.1%, respectively, suggesting that this hierarchical pattern of multiple interacting enhancers might be a common rule for gene regulation. In summary, we propose that there is a functional dichotomy in redundant enhancers. Gene regulation by regular enhancers depends on the initiator enhancers which are located closer to their target TSS and act as propagators of the regulatory signal from redundant enhancers to facilitate establishment of complex regulatory landscapes in the human genome.

## Conclusions

In this study, we identified a subset of redundant enhancers (named initiator enhancers) with DNA sequence encryption similar to self-sufficient (single) enhancers. These initiator enhancers feature distinct genomic characteristics compared to the rest of redundant enhancers: they are proximal to their target genes, they are evolutionarily conserved and they maintain a high level of chromatin contacts. GWAS and eQTLs analyses show a key role of initiator enhancers in establishing human gene regulatory programs, and the elevated level of gene expression associated with initiator enhancers indicates their function in transcriptional activation and propagation of regulatory signals from neighbouring regular enhancers. In summary, our findings reveal the existence of a critical class of enhancers playing a key role in establishing complex regulatory networks of redundant enhancers in vertebrate species.

## Additional file

Additional file 1: Supplementary material. File contains supplementary figures. (PDF 4083 kb)

## Abbreviations

auPRC: The area under the precision-recall curve
auROC: The area under the receiver operating characteristic curve
ChIP-seq: Chromatin immunoprecipitation experiments followed by sequencing
DHSs: DNase I hypersensitive sites
Encode: Encyclopedia of DNA Elements
eQTLs: Expression quantitative trait loci
GO: Gene ontology
GWAS: Genome-wide association study
SVM: Support vector machine
TAD: Topologically associating domain
TFBSs: Transcription factor binding sites
TFs: Transcription factors
TSSs: Transcription start sites
