# Supplementary material for: Dichotomy in redundant enhancers points to presence of initiators of gene regulation
Source: BMC Genomics. 2018 Dec 18;19:947. doi: 10.1186/s12864-018-5335-0 (PMC6299655; doi:10.1186/s12864-018-5335-0)
Supplement: Supplementary file 1 — Supplementary material. File contains supplementary figures. (PDF 4083 kb) [file 12864_2018_5335_MOESM1_ESM.pdf]

# Dichotomy in redundant enhancers points to presence of initiators of gene regulation

Wei Song and Ivan Ovcharenko

**Supplemental Materials**

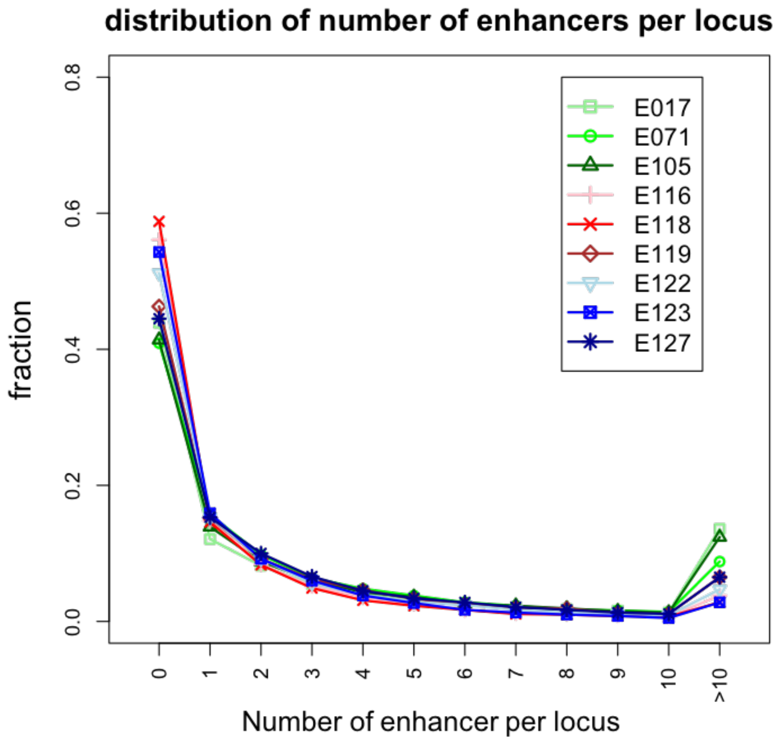


**Supplemental Figure1**. Distribution of number of enhancers per locus across nine tissues and cell lines.

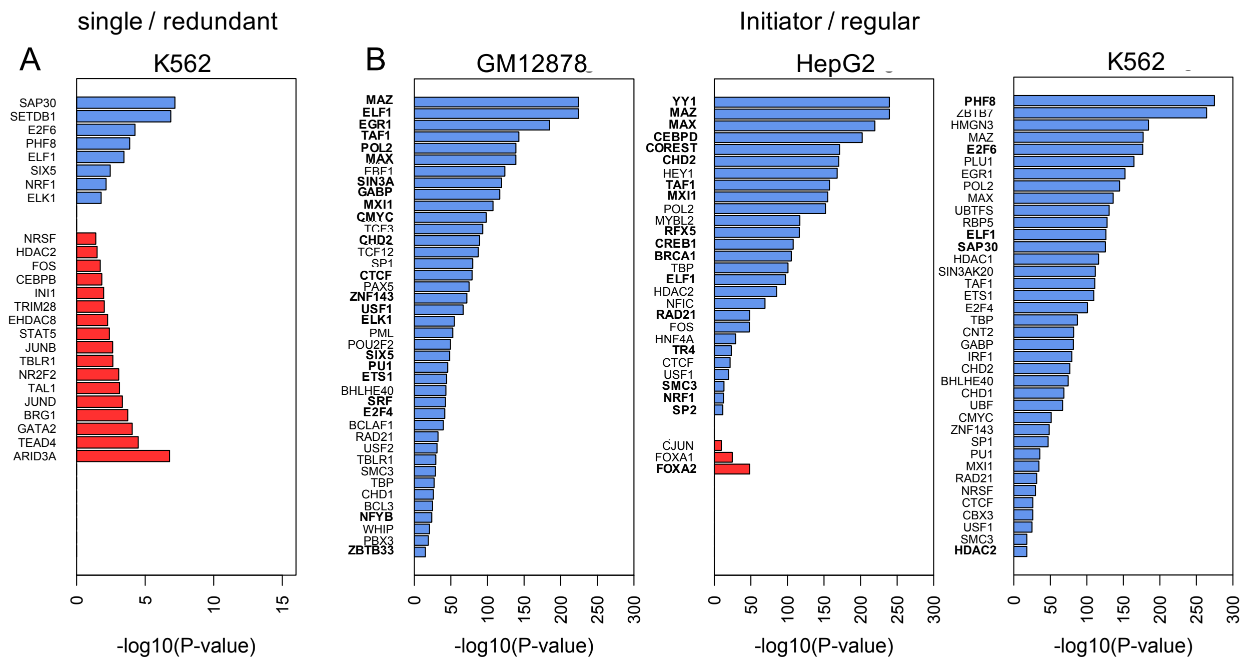


**Supplemental Figure 2**. TFBS enrichment according to significance, -log10(P-value). A) Enriched TFBSs between single and redundant enhancers in the K562 cell line. B) Enriched TFBSs between initiator and regular enhancers, in GM12878, HepG2 and K562 cell lines. Blue color shows TFBSs enriched in single or initiator enhancers, while red color shows TFBSs enriched in redundant or regular enhancers, respectively. TFs that are enriched in both single and initiator enhancers are shown in bold. P-value was calculated using the Fisher's exact test. Only TFBSs with P-value < 0.05 and enrichment fold > 1.5 are shown.

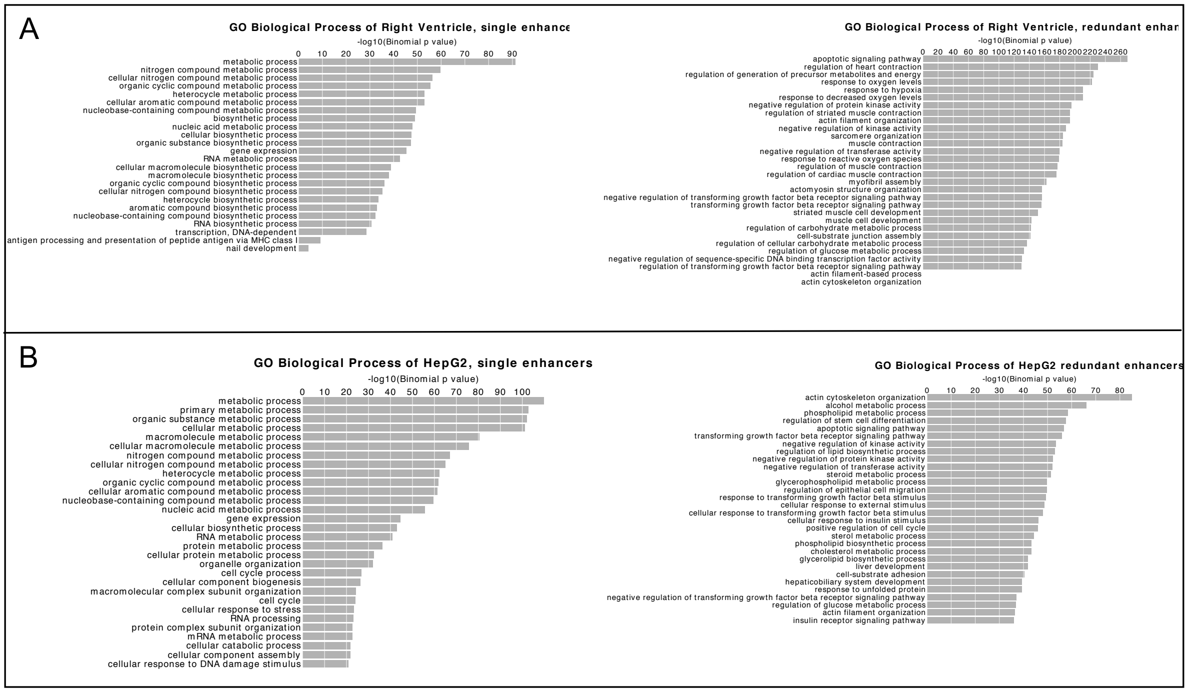


**Supplemental Figure 3.** Gene ontology enrichment analysis showing the top 30 most enriched biological processes involved in single and redundant enhancers for A) Right Ventricle and B) HepG2 cell lines.

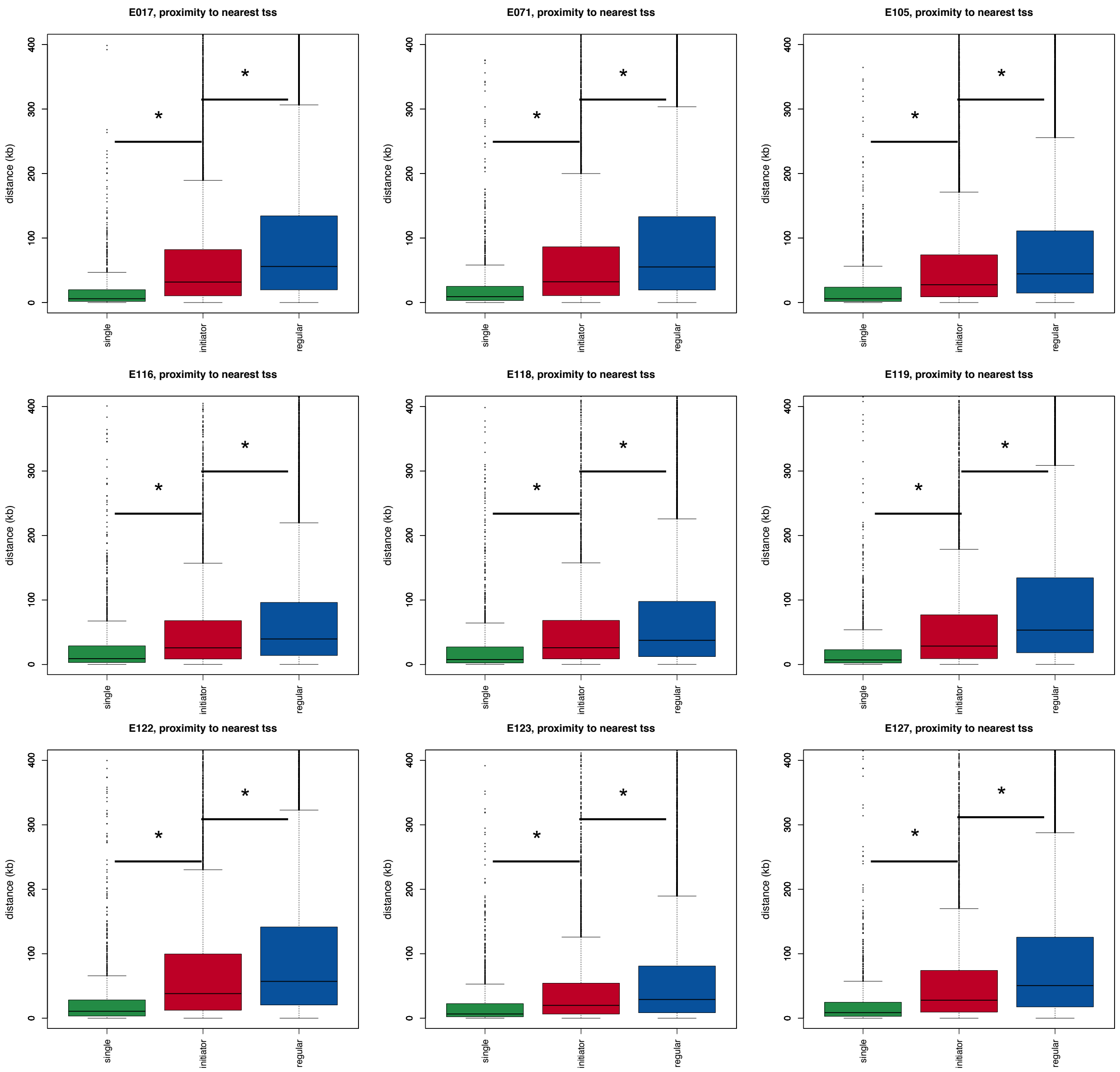


**Supplemental Figure 4.** Distribution of distance between an enhancer and its nearest TSS in nine tissues. (* means P-value < $2.2\times10^{-16}$). P-value was calculated using the Wilcoxon rank-sum test.

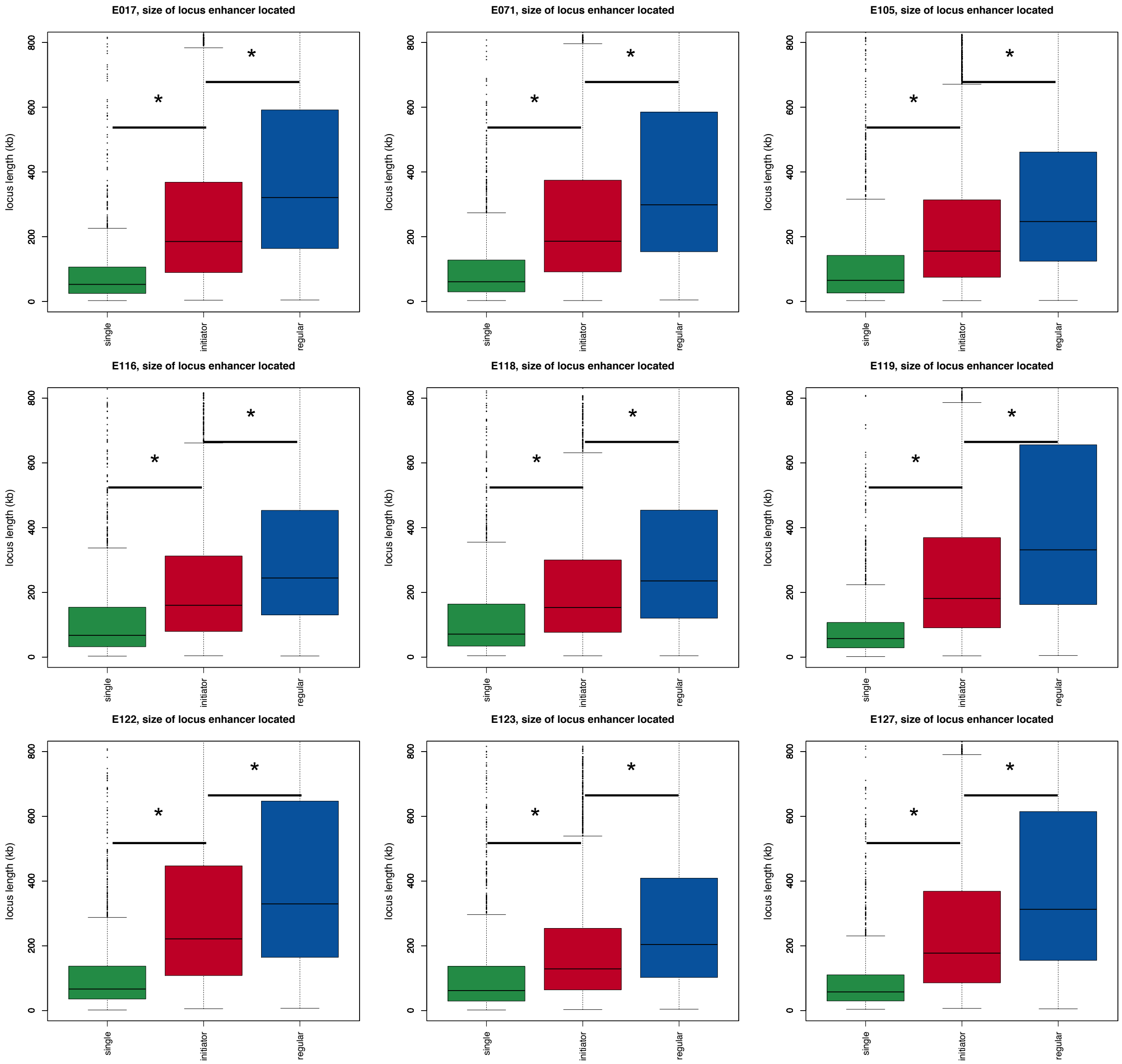


**Supplemental Figure 5.** Distribution of locus sizes in which enhancers are located (* means P-value < $2.2\times10^{-16}$). P-value was calculated using the Wilcoxon rank-sum test.

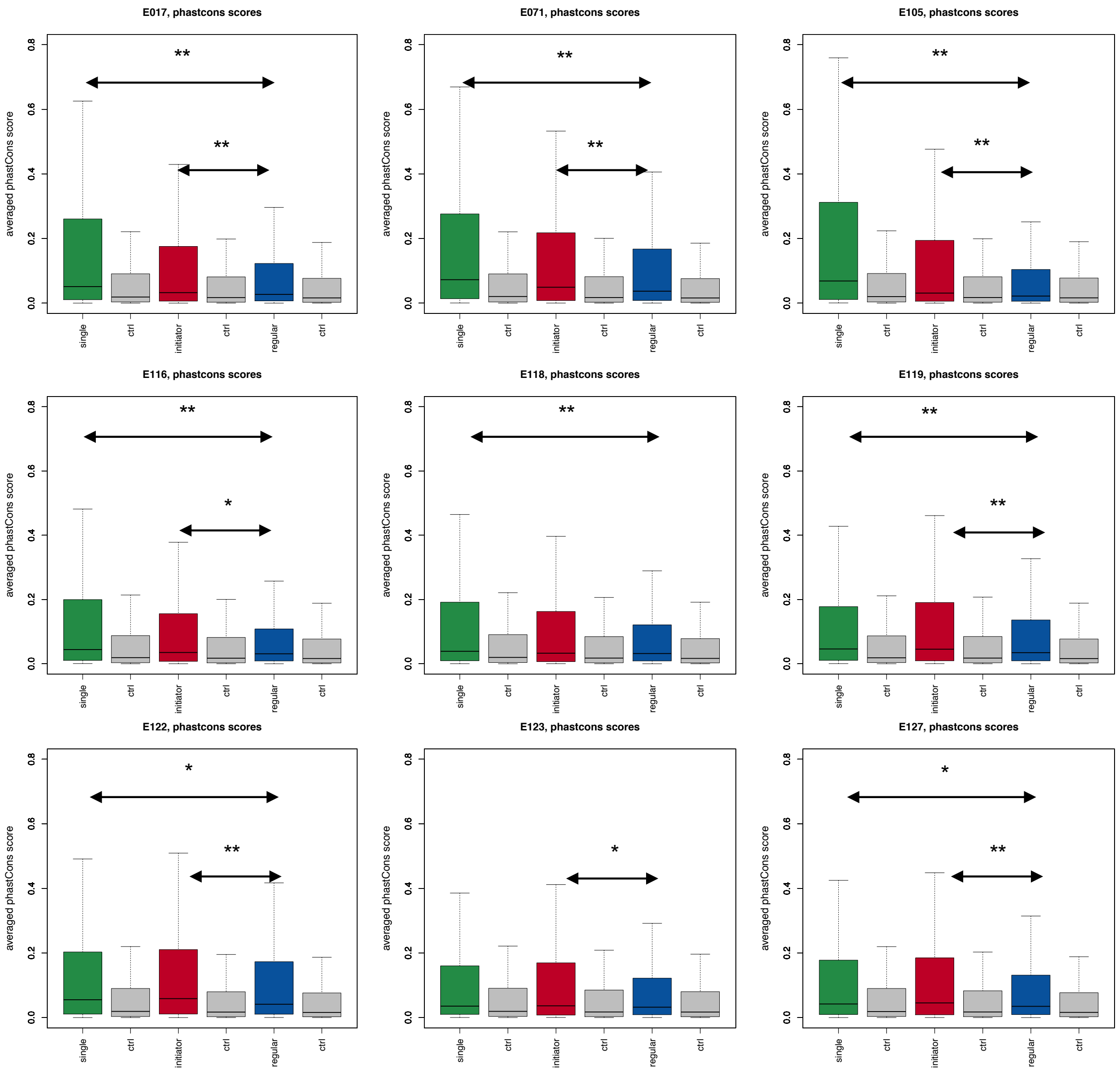


**Supplemental Figure 6.** Distribution of the average phastCons score for each category of enhancers in 9 tissues. Background phastCons scores are shown in grey. (* - P-value < 0.01 ,** - P-value < $1\times10^{-4}$). P-value was calculated using Wilcoxon rank-sum test.

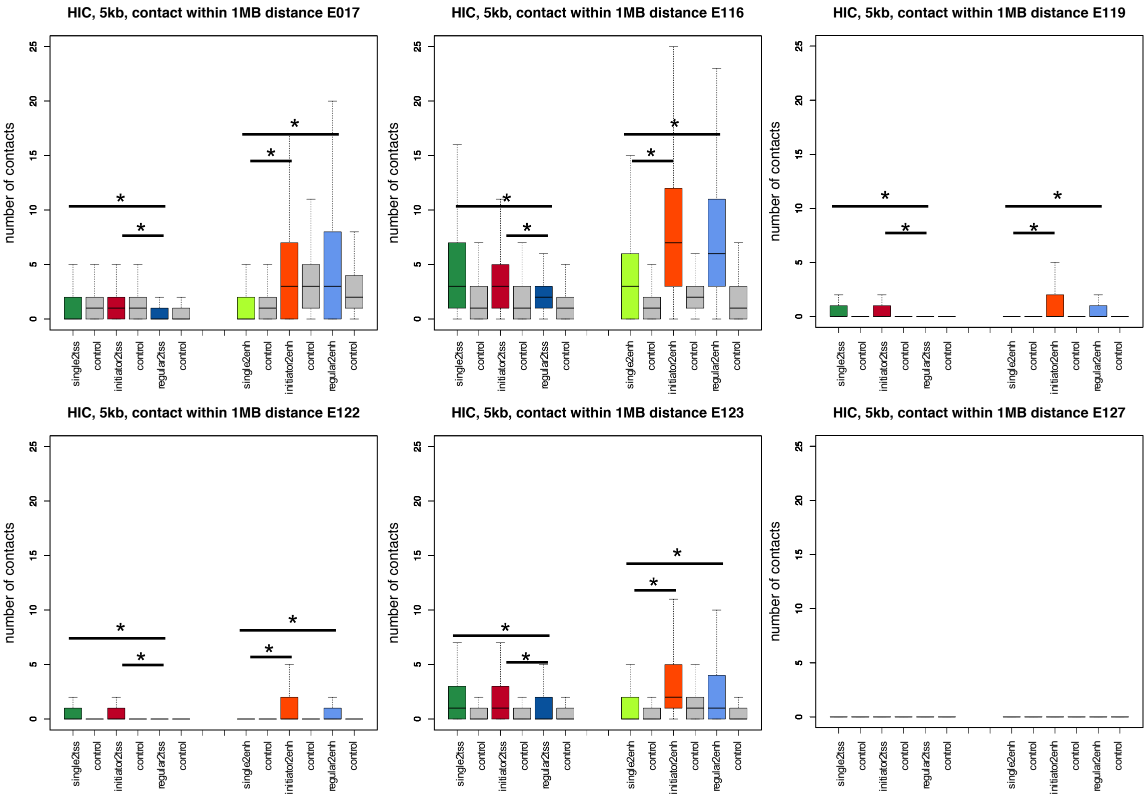


**Supplemental Figure 7.** Distribution of the number of contacts in a 1 Mb interval for each category of enhancers in 6 tissues with available Hi-C data. In each plot, the left part represents enhancer-to-TSS interactions and the right part represents enhancer-to-enhancer interactions. The background is shown as grey. (* - P-value < $1\times10^{-7}$). P-value was calculated using the Wilcoxon rank-sum test.

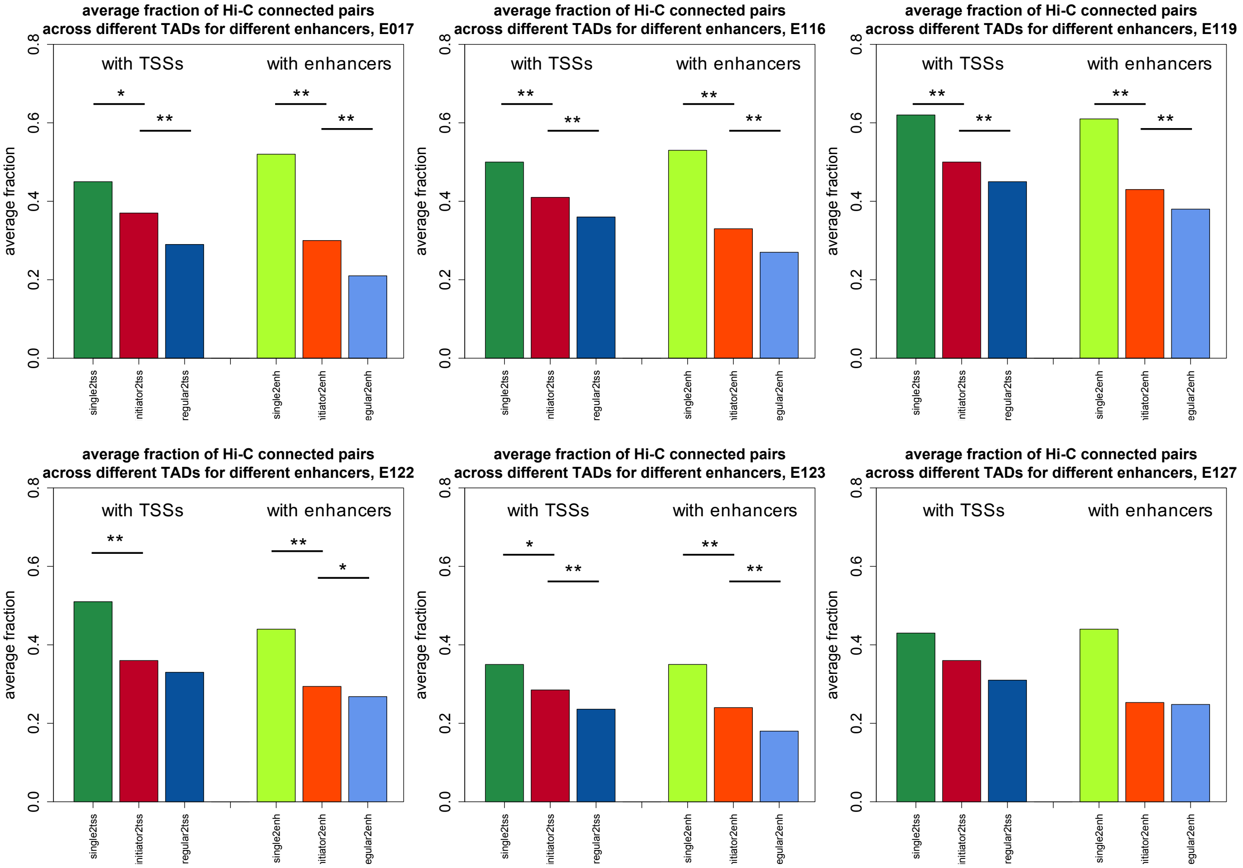


**Supplemental Figure 8.** Fraction of Hi-C connected enhancer-TSS and enhancer-enhancer pairs that are located across different TAD regions, for enhancers in each class. (* - P-value < 0.005, ** - P-value < $1\times10^{-5}$). P-value was calculated using a two sample t-test.

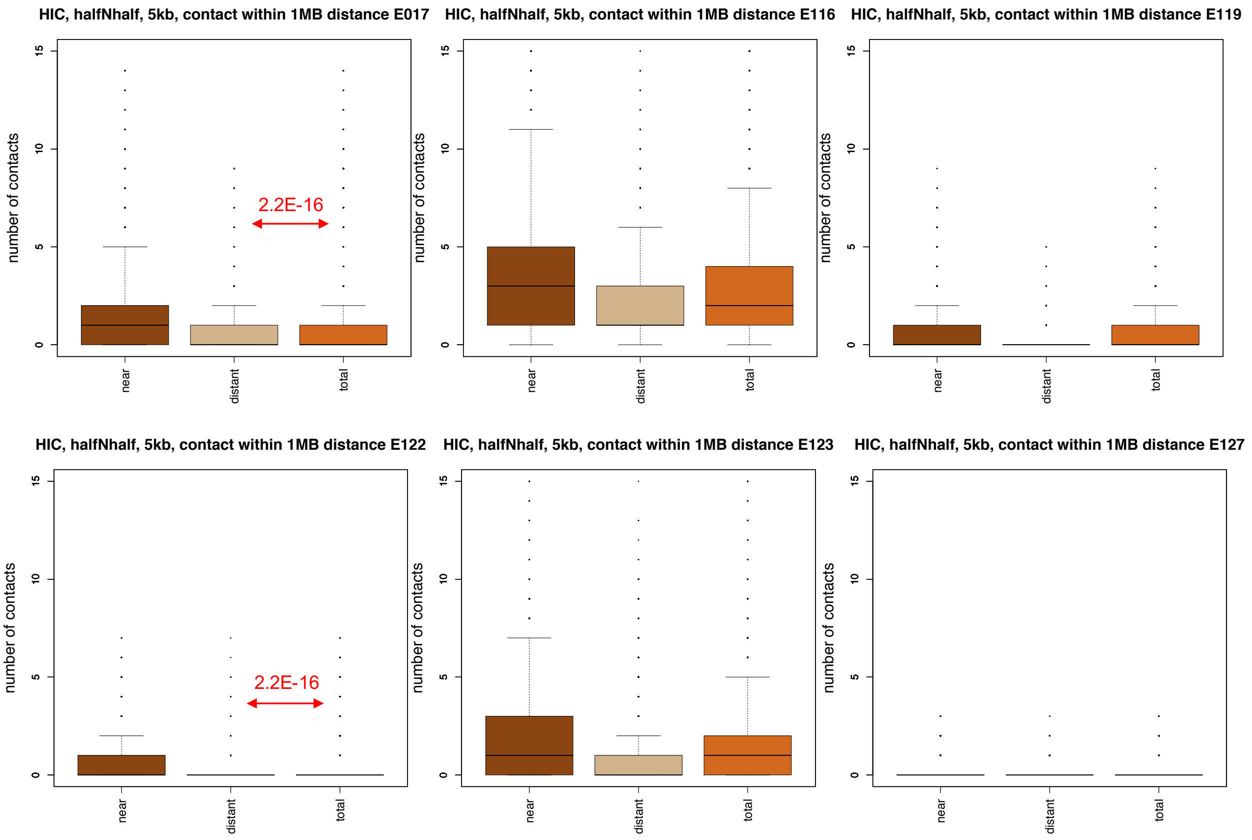


**Supplemental Figure 9.** Distribution of number of contacts for near half, distant half and total enhancers according to their distance to the nearest TSS in six tissues.

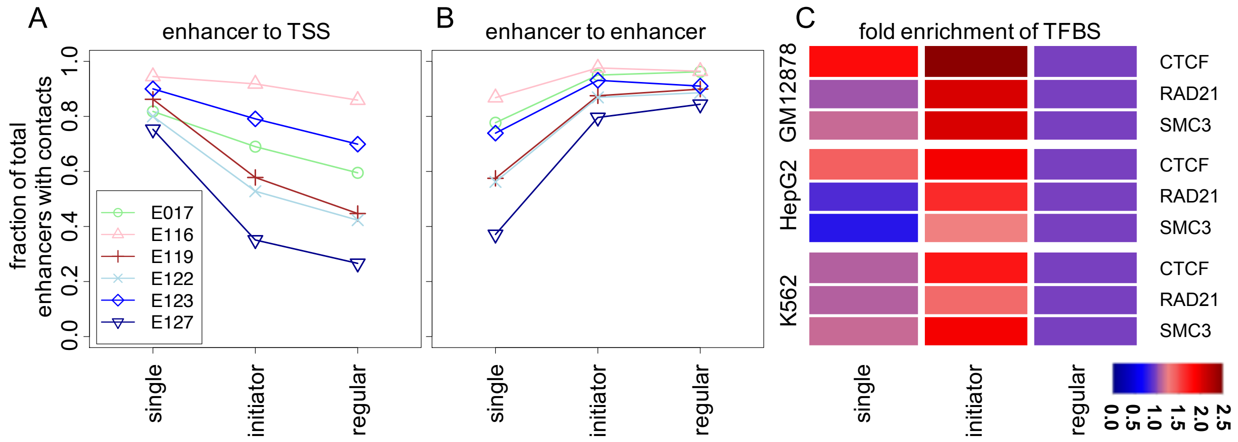


**Supplemental Figure 10.** The chromatin contacts formed by initiator enhancers. Among all the enhancers maintaining interactions with other regions, the fraction of them to interact with (A) TSS, and (B) other enhancers for the three enhancer classes in six tissues. (C) The fold-enrichment of CTCF and two cohesion components RAD21 and SMC3 for single and initiator enhancers, relative to the enrichment of regular enhancers. The fold-enrichment was calculated as the enrichment of a TFBS in all three classes divided by the enrichment of that TFBS in regular enhancers.

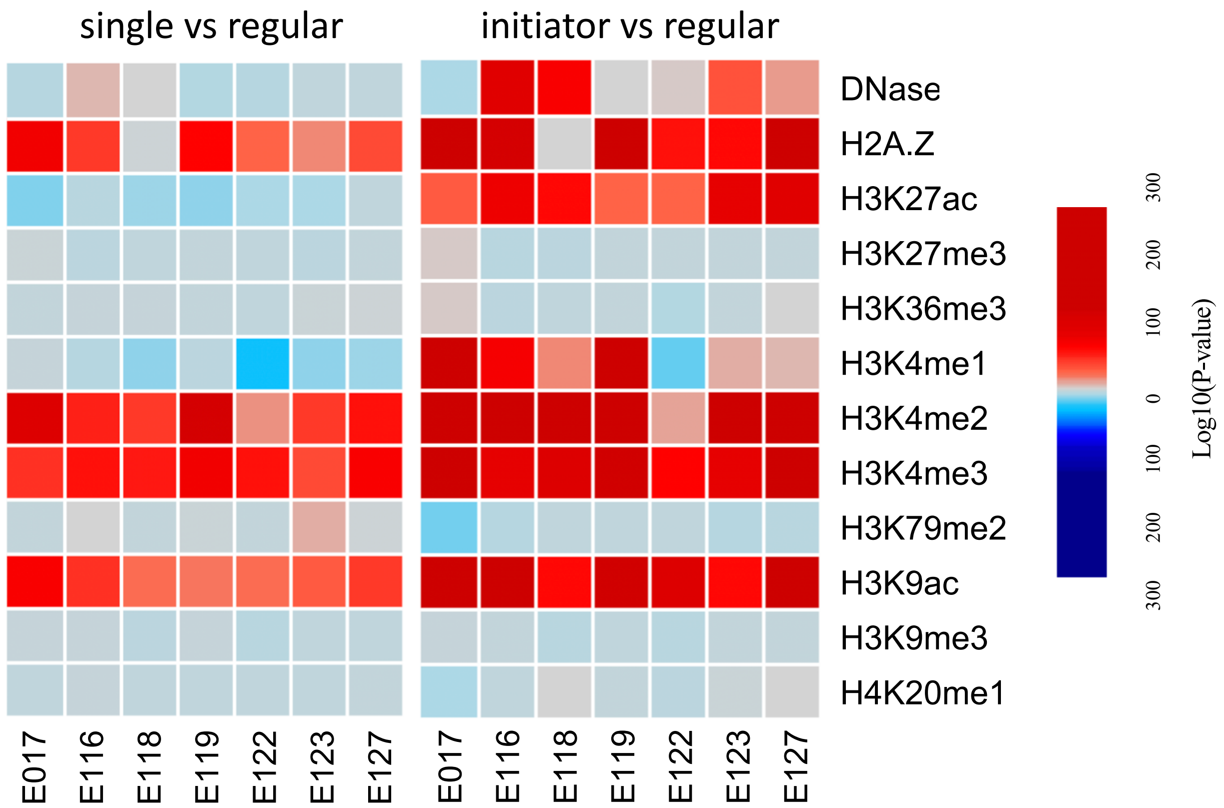


**Supplemental Figure 11.** Heatmap of signal intensities of epigenomic marks in single and initiator enhancers compared to regular enhancers across different tissues. Histone marks with fold enrichment > 1.2 are shown in the plot. Red color shows marks enriched in single or initiator enhancers. Blue color corresponds to marks enriched in regular enhancers. P-value was calculated using Wilcoxon sum rank test.

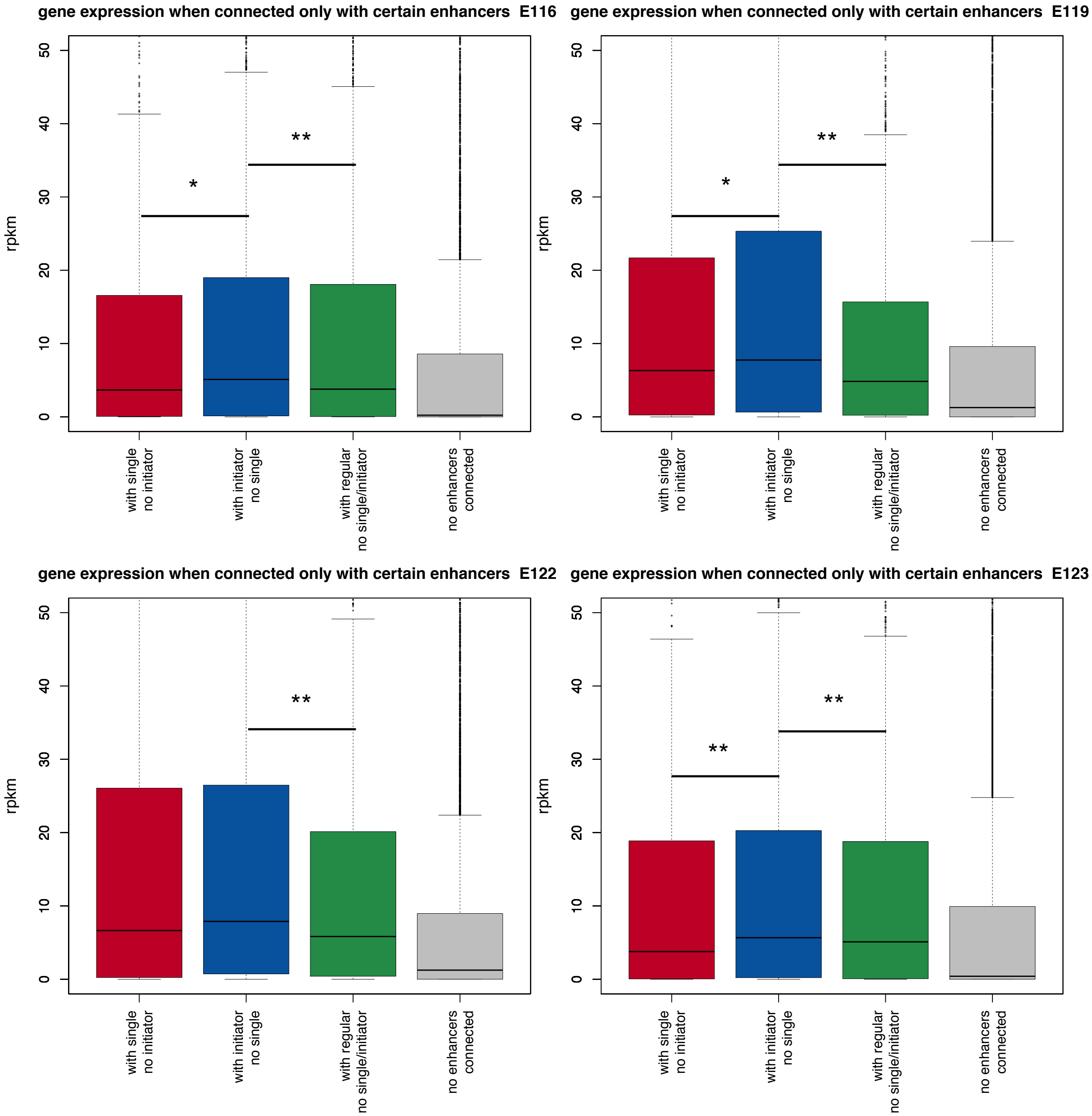


**Supplemental Figure 12.** Gene expression level associated with different classes of enhancers connected to their target genes using Hi-C contacts from GM12878, HMEC, HUVEC and K562 tissues. The labels of x-axis represent genes that 1) interact with single but not with initiator enhancers, 2) interact with initiator but not with single enhancers, 3) interact with neither single nor initiator enhancers and 4) interact with no enhancers. (* - P-value < 0.05 ,** - P-value < 0.001). P-value was calculated using the Wilcoxon rank-sum test.
